# Prevalence and Incidence of Multiple Myeloma in Urban Area in China: A National Population-Based Analysis

**DOI:** 10.3389/fonc.2019.01513

**Published:** 2020-01-24

**Authors:** Shengfeng Wang, Lu Xu, Jingnan Feng, Yang Liu, Lili Liu, Jinxi Wang, Jack Liu, Xiaojun Huang, Pei Gao, Jin Lu, Siyan Zhan

**Affiliations:** ^1^Department of Epidemiology and Biostatistics, School of Public Health, Peking University, Beijing, China; ^2^Beijing Key Laboratory of Hematopoietic Stem Cell Transplantation and Collaborative Innovation Center of Hematology, Peking University People's Hospital, Peking University Institute of Hematology, Beijing, China; ^3^Beijing Healthcom Data Technology Co. Ltd., Beijing, China; ^4^Takeda (China) International Trading Co., Ltd., Beijing, China; ^5^Innovative Center of Hematology, Soochow University, Suzhou, China; ^6^Research Center of Clinical Epidemiology, Peking University Third Hospital, Beijing, China

**Keywords:** multiple myeloma, incidence, prevalence, medical insurance, China

## Abstract

Multiple myeloma (MM) is the second most frequent malignancy of blood, and information on disease burden of MM is limited in developing countries. We aimed to estimate the prevalence and incidence of MM in China. We used data from the national urban employee and urban resident basic medical insurance from 2012 to 2016 in China. MM cases were based on the primary diagnosis (International Classification of Diseases (ICD) code, ICD for oncology, or text of diagnosis) of patients. The crude prevalence and incidence were 6.88 per 100,000 population (95% CI, 5.75–8.00) and 1.60 per 100,000 person-years (1.28–1.92), respectively. The standardized prevalence and incidence were 5.68 (5.64–5.72) and 1.15 (1.11–1.19), respectively. Overall, the rates were higher in males compared with females for prevalence (7.89 vs. 5.79, *P* < 0.05) and incidence (1.84 vs. 1.30, *P* < 0.05). Both rates increased with age, and the mean age (SD) of MM patients was 57.9 (14.4) years. Prevalence peaked between 55 and 74 years old for both genders. The incidence in women aged 55–59 had a significantly high incidence of 5.53 (4.98–6.11). The prevalence and incidence were significantly lower than those in North America, Australia, and Western Europe but were in the same range as those in Japan or Korea. MM should be one of the cancers in the spotlight from both medical and socioeconomic perspectives in low-resource but populous countries because of the incidence of more elderly MM patients in the next decade. Further research is warranted to examine the potential pathophysiologic mechanism.

## Introduction

Multiple myeloma (MM) is a neoplastic plasma cell disorder characterized by proliferation of clonal plasma cells in the bone marrow, monoclonal protein in the blood or urine, and associated organ dysfunction (1). MM is the second most frequent malignancy of the blood, which accounts for ~1% of neoplastic diseases and 13% of hematologic cancers (1, 2). During the last decades, MM has caused an increasing number of deaths globally. However, information on the epidemiology and disease burden of MM was limited, especially in the developing countries (3).

Developed countries were reported to have much higher MM incidence and prevalence than the developing countries. Three high-incidence areas around the world are North America, Australia, and Western Europe, with incidences ranging from 3 to 6 per 100,000 person-years, as well as the 5-year prevalence ranging from 7 to 14 per 100,000 population (3–5). Previous studies indicated that Asians show a relatively lower incidence than Caucasians (1, 6). The incidences reported by Japan and Korean studies were 2.0 and 1.5 per 100,000 person-years, respectively, and the corresponding 5-year prevalences were 5.4 and 3.9 per 100,000 population, respectively (4, 7). However, epidemiological studies of MM statistics were not consistent in China. Three consecutive studies conducted in Taiwan of China implied a number close to those in Japan or Korea (8–10), while the results from mainland China displayed much lower rates (4, 11). However, existing studies were subjected to only a single city (11) or calculating the rates based on the composite outcome of MM, malignant immune-proliferative diseases, and certain other B-cell lymphomas (4, 11). In addition, no further epidemiological studies were available to estimate the rates among different gender, age, and geographic groups in mainland China.

This study was conducted to provide recent estimates of the prevalence and incidence of MM in mainland China and to investigate their patterns across gender, age, and geographic groups.

## Materials and Methods

### Study Population

**Figure 1:**
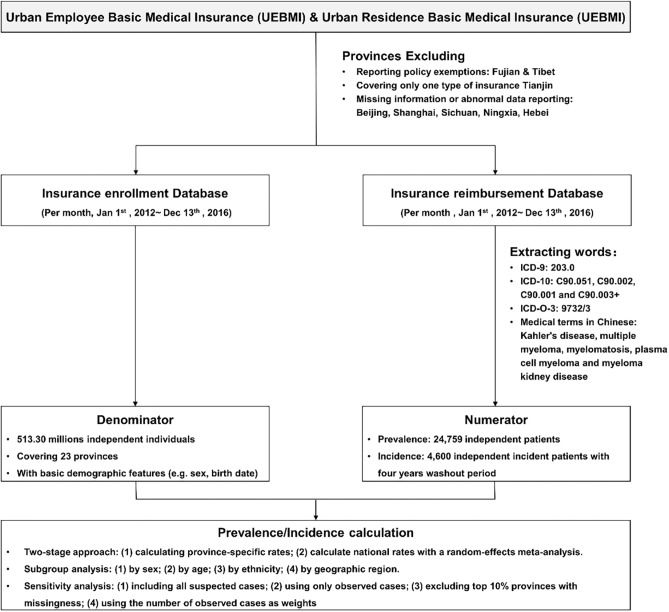
Flowchart of the study.

The data in the current study were from the national medical insurance database between January 1, 2012 and December 31, 2016 with a nationally representative population covering ~0.51 billion residents in 23 provinces (about 58.5% of urban population in China). Individuals' detailed information of the disease diagnosis was required to identify the MM admissions. Cities with no information on International Classification of Diseases (ICD) code or text of disease diagnosis were excluded. Finally, eight provinces were not included due to reporting policy exemptions (Fujian and Tibet), covering only one type of insurance (Tianjin), missing information, or having an issue of abnormal data reporting on crucial information, e.g., primary diagnosis (Beijing, Shanghai, Sichuan, Ningxia, Hebei). There are two main health insurance programs in China urban area: the Urban Employee Basic Medical Insurance (UEBMI) for urban working and retired employees and the Urban Residence Basic Medical Insurance (URBMI) for urban residents without formal employment. Until 2016, the coverage of UEBMI and URBMI in urban residents reached up to 95% (12). We used claims information from the database of UEBMI and URBMI. All claim records for this study were anonymous. The study protocol was approved by the ethical review committee of the Peking University Health Science Center (IRB. No.: IRB00001052-18012), and they waived the consent requirement. The flowchart of the study can be seen in Figure 1.

### Data Collection for the UEBMI and URBMI

Medical records will be kept in the database as long as patients provided the national insurance card for the medical service, no matter how much the patients finally paid. Both UEBMI and URBMI databases were generally updated monthly at city level. Hospital admissions for each health condition were identified based on the primary diagnoses (text of disease diagnosis or ICD code). Natural language processing was applied to normalize the text or code with a dictionary of potential MM defined by prestigious clinicians.

### MM Case Identification

MM was defined using ICD-9 (203.0), ICD-10 (C90.051, C90.002, C90.001, and C90.003+), ICD for Oncology, 3rd edition (ICD-O-3) morphologic codes (9732/3), and medical terms in Chinese including Kahler's disease, multiple myeloma, myelomatosis, plasma cell myeloma, and myeloma kidney disease. To minimize the possibility of missing MM patients, we constructed a relatively loose algorithm to extract potential MM patients with fuzzy string-matching technique, using “203.0,” “C90,” “9732,” “Kahler,” “bone marrow cancer,” and “myeloma” as key words. Diagnoses of each potential MM patient were then reviewed by two researchers independently. Exclusion criteria for patients included (1) plasma cell leukemia, (2) extramedullary plasmacytoma including plasma cell sarcoma, malignant plasma cell tumor NOS, plasmacytoma NOS, and solitary myeloma, (3) endothelial myeloma, and (4) primary myeloma. If the diagnostic items with MM contained words like “undetermined,” “uncertainty,” “?,” “possible,” and “suspicious,” the patients were also categorized as a subgroup named as “suspicious patients” used for sensitivity analysis.

### Person-Time at Risk

The date of MM onset was defined according to the date of the first MM-related claim (i.e., a claim with a diagnosis-matched MM definition) on or after January 1, 2012. The date of first MM onset also set the index year. Years prior to the index year were defined as MM-free and years following the index year were defined as prevalent MM. For incidence, the observation time began on either the date of entering the medical insurance scheme or January 1, 2016, whichever is latest for each enroller. Patients who had MM before January 1, 2016 were excluded for calculating the incidence. Person-time at risk continued to accrue until the new MM onset, disenrollment from the medical insurance scheme, or study cutoff (December 31, 2016).

### Statistical Analysis

Both rates were estimated by a commonly used two-stage approach. In the first stage, prevalence and incidence of MM were calculated in each province as follows: In the primary analysis, the denominator (*N*) to calculate the prevalence of MM was the total number of subjects in each province continuously enrolled in either UEBMI or URBMI during the study period. The numerator (*M*) was the number of patients with MM estimated in the population of denominator in each province, considering the issue of missing values. Specifically, the total enrolled population in each province can be divided into three groups: subjects not using any medical service (i.e., no records of medical claims, *N*_1_), subjects with complete information on the medical service (*N*_2_), and subjects with records of using the medical service but with missing information on the diagnosis of the medical service (*N*_3_). We observed the number of patients with MM (*M*_2_) in subjects with complete information in the medical service (*N*_2_). Considering that the reason of missing diagnosis of the medical service was generally due to the administrative issues at prefecture-level cities, we assumed that the probability of having MM was not associated with the missing status of the participants' diagnostic items. Therefore, we estimated the total number of MM cases as (*N*_2_ + *N*_3_)*M*_2_/*N*_2_. Moreover, the number of MM cases was estimated in each subgroup of different insurance type, calendar year, gender, and age group.

Incidence of MM was only estimated in 2016 and calculated by dividing the number of new MM cases by the total person-time at risk in 2016. Five provinces, including Liaoning, Guangxi, Hainan, Guizhou, and Gansu, were excluded for the estimation of incidence because of their limited time with records (<5 years). The 95% CIs of all rates were also calculated based on Poisson distribution. In the second stage, the national or regional average estimates of both rates were obtained by combining province-specific estimates using a random-effects meta-analysis.

Prevalence and incidence were also estimated by gender, age, and geographic region (East, North, North-east, North-west, South-central, and South-west) (13). Two age-adjusted rates were estimated by Segi's world population and China 2000 census data, respectively, and for comparison with other studies. Student's *t*-test for continuous variables and chi-square test for categorical variables were used in comparisons between male and female patients. All statistical tests are two-sided with *P* < 0.05 considered as statistically significant. All statistical analyses were conducted with Stata version 15.0.

### Sensitivity Analysis

Sensitivity analyses were conducted to assess the robustness of the results: (1) included all suspicious MM cases, (2) only included observed cases which are known as underestimation to assess the lower bound of the rates, and (3) excluded the top 10% of the provinces with missing diagnosis rate. In the meta-analysis, we also used the observed number of MM cases in each province as weights to consider the effect of variation in missing diagnosis rate across provinces.

## Results

From 2012 to 2016, there were ~0.51 billion enrollees in the database (Table 1). The basic population structure of UEBMI and URBMI was significantly different in gender and age distribution. A total of 24,759 had confirmed diagnosis of MM during the study period, and only 238 patients had diagnosis of suspicious MM. We therefore only focused on the confirmed MM patients in the downstream analyses. Overall, 58.68% of patients were male, and the mean ages (SD) of the male and female patients were 58.43 (14.2) and 57.0 (14.6) years, respectively (Table 2).

**Table 1:** Characteristics of populations in 23 provinces in China during 2012–2016 in the study.

		**Total**	**UEBMI**	**URBMI**
Total number (million)		513.30	233.24	280.06
Age, years	Mean (SD)	37.73 (0.001)	41.88 (0.001)	34.27 (0.001)
Age groups, *n* (%)	0–29	207.89 (40.50)	64.94 (27.84)	142.95 (51.04)
	30–34	44.90 (8.75)	30.30 (12.99)	14.60 (5.21)
	35–39	36.45 (7.10)	23.73 (10.17)	12.72 (4.54)
	40–44	40.03 (7.80)	23.59 (10.11)	16.44 (5.87)
	45–49	42.54 (8.29)	23.17 (9.93)	19.37 (6.92)
	50–54	38.93 (7.58)	20.22 (8.67)	18.71 (6.68)
	55–59	22.71 (4.42)	11.80 (5.06)	10.91 (3.90)
	60–64	25.37 (4.94)	11.85 (5.08)	13.52 (4.83)
	65–69	18.09 (3.52)	7.80 (3.34)	10.29 (3.67)
	70–74	12.46 (2.43)	5.39 (2.31)	7.07 (2.52)
	75–79	10.03 (1.95)	4.67 (2.00)	5.36 (1.92)
	80–84	7.42 (1.45)	3.30 (1.42)	4.12 (1.47)
	≥85	6.48 (1.26)	2.47 (1.06)	4.01 (1.43)
Gender, *n* (%, million)	Male	269.44 (52.49)	130.11 (55.80)	139.32 (49.75)
	Female	243.86 (47.51)	103.12 (44.21)	140.74 (50.25)
Area, *n* (%, million)	East	199.98 (38.96)	81.28 (42.39)	118.71 (34.85)
	North	21.60 (4.21)	9.08 (4.47)	12.52 (3.89)
	North-east	50.09 (9.76)	28.10 (7.85)	21.99 (12.05)
	North-west	24.15 (4.71)	10.90 (4.73)	13.25 (4.67)
	South-central	156.20 (30.43)	86.35 (24.94)	69.85 (37.02)
	South-west	61.28 (11.94)	17.54 (15.62)	43.74 (7.52)

*SD, standard deviation; UEBMI, urban employee basic medical insurance; URBMI, urban resident basic medical insurance*.

*East area contains Jiangsu, Zhejiang, Anhui, Jiangxi, and Shandong (five provinces). North area contains Shanxi, Inner Mongolia (two provinces). North-east contains Liaoning, Jilin, and Heilongjiang (three provinces). North-west contains Shaanxi, Qinghai, Gansu, and Xinjiang (four provinces). South-central contains Henan, Hubei, Hunan, Guangdong, Guangxi, and Hainan (six provinces). South-west contains Chongqing, Guizhou, and Yunan (three provinces)*.

**Table 2:** Characteristics for patients with multiple myeloma in 23 provinces in China during 2012–2016 in the study.

**Characteristic**		**Total**	**Male**	**Female**	***P*-value**
Number		24,759	13,358	9,417	
Age, years					<0.001
	Mean (SD)	57.91 (14.38)	58.43 (14.24)	57.04 (14.55)	
Age group, *n* (%)					<0.001
	0–29	1,023 (4.13)	567 (4.24)	456 (4.84)	
	30–34	489 (1.98)	270 (2.02)	217 (2.30)	
	35–39	811 (3.28)	407 (3.05)	403 (4.28)	
	40–44	1,372 (5.54)	771 (5.77)	597 (6.34)	
	45–49	2,152 (8.69)	1,229 (9.20)	915 (9.72)	
	50–54	2,187 (8.83)	1,245 (9.32)	927 (9.84)	
	55–59	3,371 (13.62)	1,930 (14.45)	1,419 (15.07)	
	60–64	3,527 (14.25)	2,072 (15.51)	1,429 (15.17)	
	65–69	3,002 (12.12)	1,794 (13.43)	1,181 (12.54)	
	70–74	2,606 (10.53)	1,593 (11.93)	979 (10.40)	
	75–79	1,662 (6.71)	1,025 (7.67)	615 (6.53)	
	80–84	596 (2.41)	362 (2.71)	220 (2.34)	
	≥85	151 (0.61)	91 (0.68)	59 (0.63)	
Year, *n* (%)					0.395
	2012	3,754 (15.16)	1,914 (14.33)	1,374 (14.59)	
	2013	4,396 (17.76)	2,371 (17.75)	1,706 (18.12)	
	2014	4,481 (18.10)	2,509 (18.78)	1,696 (18.01)	
	2015	5,269 (21.28)	2,872 (21.50)	1,975 (20.97)	
	2016	6,859 (27.70)	3,692 (27.64)	2,666 (28.31)	
Area, *n* (%)					0.001
	East	10,939 (44.18)	5,868 (43.93)	4,077 (43.29)	
	North	878 (3.55)	521 (3.90)	336 (3.57)	
	North-east	3,338 (13.48)	1,664 (12.46)	1,291 (13.71)	
	North-west	787 (3.18)	489 (3.66)	270 (2.87)	
	South-central	5,985 (24.17)	3,154 (23.61)	2,295 (24.37)	
	South-west	2,832 (11.44)	1,662 (12.44)	1,148 (12.19)	

*SD, standard deviation*.

*A total of 1,984 patients had missing information in gender. Area is defined as in Table 1*.

### Prevalence

**Figure 2:**
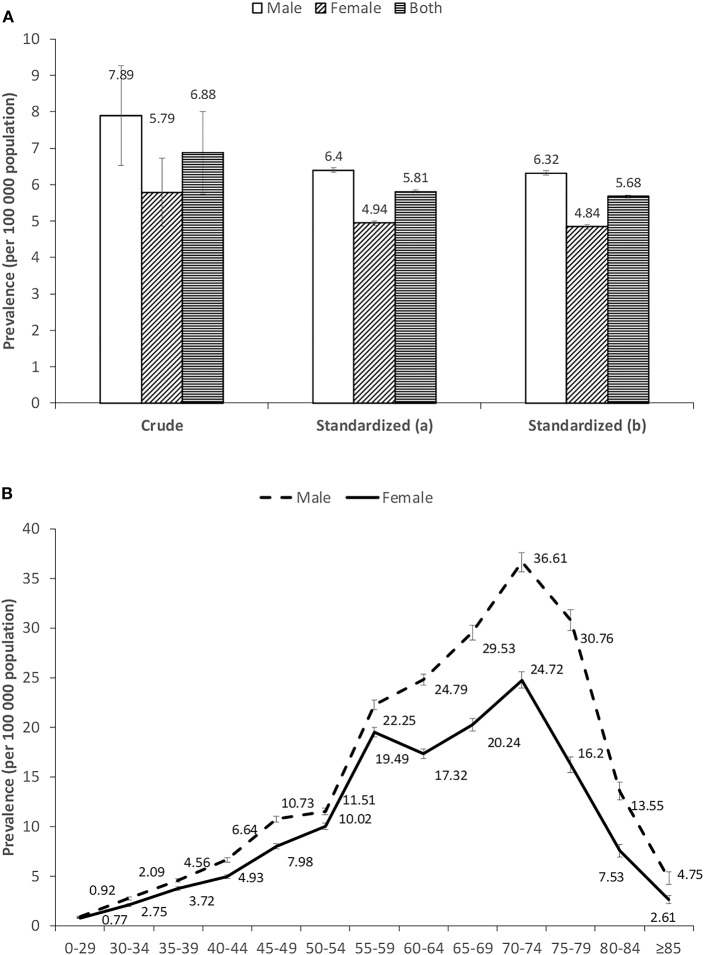
**(A,B)** Prevalence of multiple myeloma in China during 2012–2016. Standardized (a) and standardized (b) means age-standardized rates estimated by Segi's world population and China 2000 census data, respectively.

The national prevalence was 6.88 per 100,000 population (95% CI; 5.75–8.00) (Figure 2). Prevalence was always higher in males than in females, i.e., 7.89 per 100,000 population (95% CI; 6.52–9.26) for males and 5.79 (95% CI; 4.85–6.73) for females, respectively (Figure 2). The prevalence varied by age, with a bell shape peaking between 55 and 74 years old in both genders (Figure 2). The highest rate was in patients aged 70–74 years old for each gender, with values of 36.61 per 100,000 population and 24.72 per 100,000 population for males and females, respectively (eTable 1). Comparing patients less and more than 60 years old, the prevalence more than doubled and the gender difference was enlarged in the older age group (eTable 1). Northern China and eastern China had relatively higher rates of MM that the rest of the areas (eTables 2, 3).

### Incidence

**Figure 3:**
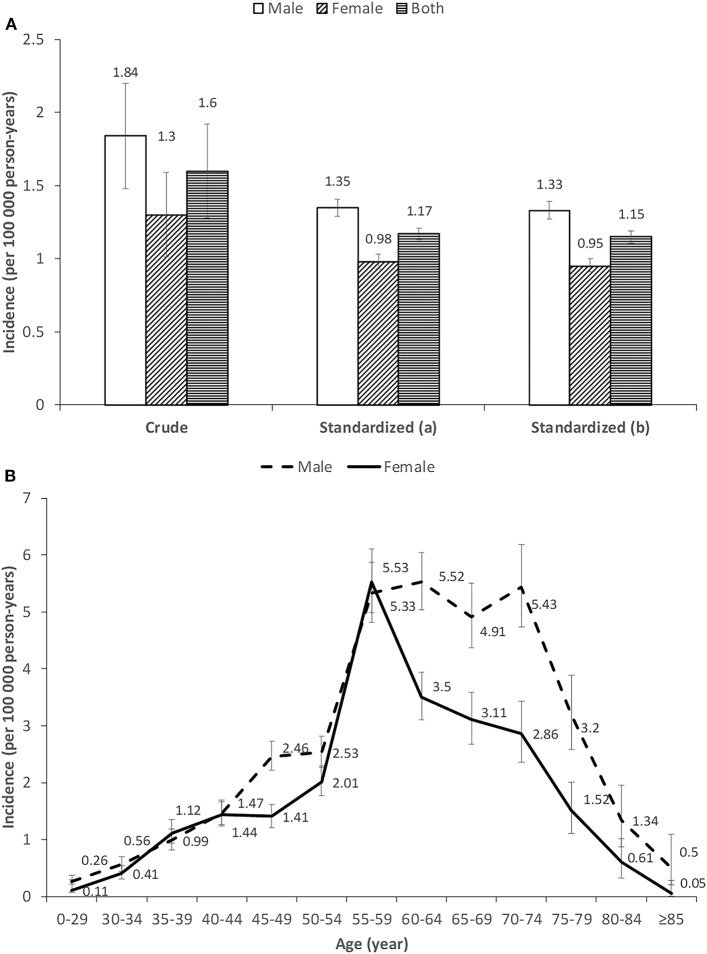
**(A,B)** Incidence of multiple myeloma in China in 2016. Standardized (a) and standardized (b) means age-standardized rates were estimated by Segi's world population and China 2000 census data, respectively.

The national incidence of MM in 2016 was 1.60 per 100,000 person-years (95% CI; 1.28–1.92) (Figure 3). The incidence rate was 1.84 per 100,000 person-years (95% CI; 1.48–2.20) for males and 1.30 (95% CI; 1.01–1.59) for females, respectively (Figure 3). The incidence was also shown to sharply increase after the age of 55 years old for both genders. However, the incidence of MM remained high until the age of 70–74 years old for males, whereas there was an immediate decline thereafter for females (Figure 3). Compared with patients younger than 60 years old, the incidence of elders was more than doubled. The gender difference was enlarged in the older age group (eTable 4). North-eastern and eastern China had slightly higher rates of MM than the rest of the areas (eTables 2, 3).

### The Standardized Rates

From 2012 to 2016, the overall average prevalence for Segi's world standard population (WSR) was 5.68 per 100,000 population (95% CI, 5.64–5.72), with 6.32 (95% CI, 6.26–6.38) in males and 4.84 (95% CI, 4.78–4.90) in females, respectively. Meanwhile, the incidence standardized by WSR in 2016 was 1.15 per 100,000 person-years (95% CI, 1.11–1.19), with 1.33 (95% CI, 1.27–1.39) in males and 0.95 (95% CI, 0.91–1.00) in females, respectively (Table 3).

**Table 3:** Standardized prevalence and incidence of multiple myeloma in China during 2012−2016 (units: /100 000 population for prevalence; /100,000 person-years for incidence).

	**Standardized prevalence** **(95% CI)**	**Standardized incidence** **(95% CI)**
**Standardized by china 2000 population census data**		
Male	6.40 (6.34–6.46)	1.35 (1.29–1.41)
Female	4.94 (4.88–5.00)	0.98 (0.93–1.03)
Total	5.81 (5.77–5.85)	1.17 (1.13–1.21)
**Standardized by segi's world standard population**		
Male	6.32 (6.26–6.38)	1.33 (1.27–1.39)
Female	4.84 (4.78–4.90)	0.95 (0.91–1.00)
Total	5.68 (5.64–5.72)	1.15 (1.11–1.19)

*CI, confidence interval*.

The prevalence standardized by China 2000 population census data was 5.81 per 100,000 population (95% CI, 5.77–5.85), with 6.40 (95% CI, 6.34–6.46) in males and 4.94 (95% CI, 5.77–5.85) in females, respectively. Meanwhile, the corresponding incidence in 2016 was 1.17 per 100,000 person-years (95% CI, 1.13–1.21), with 1.35 (95% CI, 1.29–1.41) in males and 0.98 (95% CI, 0.93–1.03) in females, respectively.

### Sensitivity Analysis

Broadly similar estimations were obtained if we included all suspicious MM cases. The lower bounds of the overall rates was 3.47 (95% CI, 2.73–4.20) for the prevalence and 0.85 (95% CI, 0.70–0.99) for the incidence if we used only observed cases which are known to be underestimated. Different methods of meta-analysis provided a slightly higher estimation of the prevalence, whereas they gave a similar estimation of the incidence (eTable 5).

## Discussion

In this national study, we elucidated three primary findings. Firstly, during 2012–2016, the estimated average age-adjusted prevalence in mainland China was 5.68 per 100,000 population, and the incidence was 1.15 per 100,000 person-years in 2016. These estimations showed that both prevalence and incidence were significantly lower than those in North America, Australia, and Western Europe (3, 4), but were in the same range as those in Japan or Korea (4, 7). The current rates were more than doubled than in GLOBAOCAN 2012, with incidence of 0.56 per 100,000 person-years and prevalence of 1.2 per 100,000 population, but closer to the recent results of GLOBOCAN 2018 with the incidence of 0.92 per 100,000 person-years and the prevalence of 2.1 per 100,000 population for China (4, 14). Our current results were still lower than the average incidence of 2.21 per 100,000 person-years from 2011 to 2012 in Taiwan, which has a larger proportion of elderly population than in the mainland (9, 10). Although Asians including Chinese show a relatively lower incidence than Caucasians (4, 15), the actual number of incidents of multiple myeloma patients in Asia was still higher because of the huge population. Males were more likely to have MM than females, with a 1.40-fold increased risk. This was consistent with previous studies in Asian and other races (2, 8–10, 16).

In our study, the prevalence of MM varied by the geographic areas of China. It is noteworthy that northern China and eastern China presented relatively higher rates of MM than the rest of the areas. Differences in genetic background, culture, climate, and lifestyle patterns might all contribute to disparities across regions (3, 8, 17–19). For example, a potential explanation for the lower rates in the southern areas might be the relatively lower height of their residents. Previous studies reported a modest increased risk for taller individuals (20–23). Further investigation is needed to investigate and find the causes.

Secondly, the mean age of the Chinese patients with MM was 58, which was about 10 years younger than that of Caucasians. Three quarters of patients were diagnosed above the age of 49 years old. This was consistent with previous epidemiological studies in China (9, 24). This age was even slightly younger than those of patients from Japan, Korea, and Taiwan of China (24). Ethnic disparity might be one potential reason for this wide gap (3), considering the fact that bone geometry, quality, and strength differ between Asians and Caucasians (25, 26). Another explanation that should also be noted is that the age of diagnosis for MM seems to be closely related to the mean life expectancy in corresponding regions (24). Indeed the current age of diagnosis in China is very similar to that in USA about 20 years ago (2). Therefore, on the background of population aging in developing countries including China, more elderly MM patients would appear in developing countries in the future. Consequently, MM will be one of the cancers in the spotlight in those low-resource but populous countries from both medical and socioeconomic perspectives.

Thirdly, a dramatic increase of MM incidence was observed for Chinese females in the age group of 55–59 years old. There was a clear difference between males and females for the pattern of incidence, i.e., the incidence of MM remained high until age 70–74 years old for males, whereas there was an immediate decline thereafter for females. Similar creeping up was also observed in America, Europe, Australia (15, 27, 28). However, the MM risk for western populations continued to rise thereafter and will peak until 10–15 years later (8, 10, 15, 27, 28). No specific incidence data for women aged 55–59 years old were available for other Asian regions. Two aforementioned studies in Taiwan did not subdivide this age group (8, 10). This dramatic increase of the specific age group is worthy to note considering that this age group is of special interest due to menopause for most Chinese women soon after. This may suggest a role of estrogen in hematological malignancies including MM (29). Various interactions exist for reproductive hormone with the immune system in females (30). The bone marrow microenvironment is a reservoir of immune cells, while MM cell proliferation and survival rely on factors produced by cells of the bone microenvironment. Further research is warranted to examine its potential pathophysiologic mechanism.

This study has several strengths. This is a large, national representative sample of Chinese mainland population, ensuring the estimation of both rates of a rare disease. It allowed us to not only provide the overall estimation of the both rates but also explore age and gender patterns of the rates as well as the geographic variations across the countries. This study also has several limitations. First, the diverse missing proportion of diagnosis-related variables could have affected the estimates. However, several sensitivity analyses were conducted to explore the potential influences of the estimations. Especially, the lower bounds of the rates were presented using only observed cases of MM, which could facilitate the interpretation of the findings. Secondly, the basic medical insurance database did not have detailed information regarding biopsies, laboratory data, tumor stage, and cause of death. The extracted patients with MM were generally diagnosed cases. It precluded the possibility to confirm unknown MM cases from laboratory tests. Thirdly, the new MM cases for the estimation of incidence were defined as 4-year disease-free before the index claim, which may not be sufficient. However, a Myeloma Network study reported that the median overall survival of MM patients in Asian countries including China was 47 months (24), which was consistent with our results that the prevalence to incidence ratio was about 4.9. Finally, a few provinces were excluded; we could not describe the features of the excluded populations due to lacking information. In addition, certain urban populations such as college students and military soldiers were not included in the study because they have different types of medical insurance. Their exclusion could have affected the estimates.

The prevalence and incidence of MM were significantly lower than those in North America, Australia, and Western Europe but were in the same range as those in Japan or Korea. Chinese MM patients were younger. A significantly high incidence of MM was observed for Chinese women in the age group of 55–59 years old just after menopause. Further research is warranted to examine the potential pathophysiologic mechanism.

## Data Availability Statement

The datasets analyzed in this article are not publicly available. Requests to access the datasets should be directed to Siyan Zhan, siyan-zhan@bjmu.edu.cn.

## Ethics Statement

The study protocol was approved by the ethical review committee of the Peking University Health Science Center (IRB. No.: IRB00001052-18012), and they waived the consent requirement.

## Author Contributions

SZ, XH, JLi, SW, JLu, YL, and PG: study conception and design. JW, LX, JF, SW, JLu, YL, LL, PG, and SZ: acquisition, analysis, or interpretation of data. SW and PG: draft of the manuscript. SW, LX, JF, and PG: statistical analysis. XH, SZ, and PG: supervision.

### Conflict of Interest

JL was employed by the company Takeda (China) International Trading Co., Ltd and only contributed to the concept of the study. The remaining authors declare that the research was conducted in the absence of any commercial or financial relationships that could be construed as a potential conflict of interest.
