# Supplementary material for: Prevalence and Incidence of Multiple Myeloma in Urban Area in China: A National Population-Based Analysis
Source: Front Oncol. 2020 Jan 24;9:1513. doi: 10.3389/fonc.2019.01513 (PMC6993203; doi:10.3389/fonc.2019.01513)
Supplement: Supplementary file 1 [file Data_Sheet_1.pdf]

Supplementary Material

eTable 1 Crude prevalence of multiple myeloma in 23 provinces in China during 2012--2016, grouped by gender, age-group, and location. (Units: /100 000 population)

	Male			Female		
	**# of Person-year**	**# of cases[a]**	**Prevalence (95% CI)**	**# of Person-year**	**# of cases[a]**	**Prevalence (95% CI)**
Total	6154.2	50699	8.24 (8.17-8.31)	5610.5	34842	6.21 (6.15-6.28)
Age group						
0-29	2467.54	2271	0.92 (0.88-0.96)	2174.53	1669	0.77 (0.73-0.81)
30-34	528.72	1453	2.75 (2.61-2.89)	477.23	996	2.09 (1.96-2.22)
35-39	463.39	950	4.56 (4.37-4.76)	432.96	1611	3.72 (3.54-3.91)
40-44	531.5	3527	6.64 (6.42-6.86)	501.91	2475	4.93 (4.74-5.13)
45-49	524.04	5621	10.73 (10.45-11.01)	488.02	3895	7.98 (7.73-8.24)
50-54	435.9	5017	11.51 (11.19-11.83)	391.16	3918	10.02 (9.71-10.33)
55-59	325.88	7249	22.25 (21.74-22.76)	285.43	5563	19.49 (18.98-20.01)
60-64	299.98	7436	24.79 (24.23-25.36)	276.49	4790	17.32 (16.84-17.82)
65-69	208.07	6145	29.53 (28.80-30.28)	193.56	3918	20.24 (19.61-20.89)
70-74	145.57	5329	36.61 (35.63-37.60)	139.86	3457	24.72 (23.90-25.56)
75-79	110.22	3390	30.76 (29.73-31.81)	112.00	1814	16.20 (15.46-16.96)
80-84	68.99	935	13.55 (12.70-14.45)	76.71	578	7.53 (6.93-8.17)
>=85	44.4	211	4.75 (4.13-5.44)	60.66	158	2.61 (2.21-3.04)

Area						
East	2601.08	26324	10.12 (10.00-10.24)	2397.65	17743	7.40 (7.29-7.51)
North	252.28	3080	12.21 (11.78-12.65)	247.96	2288	9.23 (8.85-9.61)
North-East	671.83	5445	8.10 (7.89-8.32)	663.43	3821	5.76 (5.58-5.95)
North-West	236.85	1286	5.43 (5.14-5.73)	215.28	934	4.34 (4.06-4.63)
South-Central	1608.53	9863	6.13 (6.01-6.25)	1369.5	6875	5.02 (4.90-5.14)
South-West	783.63	4701	6.00 (5.83-6.17)	716.68	3182	4.44 (4.29-4.60)

[a] Including all observed cases and predicted number of cases in people missing diagnostic information.

eTable 2 Prevalence and incidence of multiple myeloma in different regions of China during 2012-2016.

	Prevalence (per 100 000 population)			Incidence (per 100 000 person-years)		
**Groups**	**# of 10^5 population**	**# of Case [a]**	**Rate (95%CI)**	**# of 10^5 person-years**	**# of incident Case [a]**	**Rate (95%CI)**
East[b]	4998.73	44067	8.82 (8.73-8.90)	1450.05	2573	1.77 (1.71-1.84)
North	500.24	5368	10.73 (10.45-11.02)	162.57	151	0.93 (0.79-1.09)
North-East	1335.26	9265	6.94 (6.80-7.08)	149.11	263	1.76 (1.56-1.99)
North-West	452.13	2219	4.91 (4.71-5.12)	145.06	171	1.18 (1.01-1.37)
South-Central	2978.04	16738	5.62 (5.54-5.71)	779.18	1145	1.47 (1.39-1.56)
South-West	1500.31	7883	5.25 (5.14-5.37)	425.35	297	0.70 (0.62-0.78)

[a] Including all observed case and predicted number of cases in people missing diagnostic information.

[b] Area was defined as in Table 1.

.

eTable 3 Crude prevalence and incidence of multiple myeloma in China during 2012-2016, grouped by provinces (Units: /100 000 population for prevalence; /100 000 person-years for incidence)

	Prevalence			Incidence		
**Groups**	**# of 10^5 population**	**# of Case**	**Rate (95%CI)**	**# of 10^5 person-years**	**# of incident Case**	**Rate (95%CI)**
Shanxi	214.22	3864	18.04 (17.47-18.62)	69.45	49	0.70 (0.52-0.93)
Inner Mongoria	286.01	1503	5.26 (4.99-5.53)	93.11	102	1.10 (0.89-1.33)
Liaoning[a]	852.17	7367	8.65 (8.45-8.84)	-	-	-
Jilin	373.63	1318	3.53 (3.34-3.72)	86.88	67	0.77 (0.60-0.98)
Heilongjiang	109.46	580	5.30 (4.88-5.75)	62.25	196	3.15 (2.72-3.62)
Jiangsu	1338.82	12590	9.40 (9.24-9.57)	298.49	377	1.26 (1.14-1.40)
Zhejiang	832.67	10087	12.11 (11.88-12.35)	244.83	368	1.50 (1.35-1.66)
Anhui	335.78	3408	10.15 (9.81-10.50)	83.54	167	2.00 (1.71-2.33)
Jiangxi	349.02	2945	8.44 (8.14-8.75)	85.82	272	3.17 (2.80-3.57)
Shandong	2142.45	15037	7.02 (6.91-7.13)	737.37	1388	1.88 (1.78-1.98)
Henan	308.77	1976	6.40 (6.12-6.69)	111.49	360	3.23 (2.90-3.58)
Hubei	335.92	4529	13.48 (13.09-13.88)	119.18	365	3.06 (2.76-3.39)
Hunan	435.5	2082	4.78 (4.58-4.99)	113.81	145	1.27 (1.08-1.50)
Guangdong	1686.63	7489	4.44 (4.34-4.54)	434.72	275	0.63 (0.56-0.71)
Guangxi[a]	89.9	480	5.34 (4.87-5.84)	-	-	-

Groups	Prevalence			Incidence		
	# of 10^5 population	# of Case	Rate (95%CI)	# of 10^5 person-years	# of incident Case	Rate (95%CI)
Hainan[a]	121.33	181	1.49 (1.28-1.73)	-	-	-
Chongqing	997.23	4983	5.00 (4.86-5.14)	318.74	233	0.73 (0.64-0.83)
Guizhou[a]	65.94	202	3.06 (2.66-3.52)	-	-	-
Yunnan	437.14	2699	6.17 (5.94-6.41)	106.62	64	0.60 (0.46-0.77)
Shaanxi	208.89	1113	5.33 (5.02-5.65)	74.34	48	0.65 (0.48-0.86)
Gansu[a]	36.25	217	5.99 (5.22-6.84)	-	-	-
Qinghai	64.78	306	4.72 (4.21-5.28)	16.62	30	1.79 (1.22-2.58)
Xinjiang	142.2	583	4.10 (3.77-4.45)	54.11	93	1.72 (1.39-2.11)
Total	11764.7	85540	7.27 (7.22-7.32)	3111.34	4600	1.48 (1.44-1.52)

[a] Five provinces (Liaoning, Guangxi, Hainan, Guizhou, and Gansu) were excluded due to the time ranges <5 years.

eTable 4 Crude incidence of multiple myeloma in 18 provinces in China in 2016, grouped by gender, age-group, and location (Units: /100 000 person-years)

	Male			Female		
	**# of Person-year**	**# of new cases**	**Incidence (95% CI)**	**# of Person-year**	**# of new cases**	**Incidence (95% CI)**
Total	1616.96	2741	1.70 (1.63-1.76)	1494.37	1859	1.24 (1.19-1.30)
Age group						
0-29	636.98	166	0.26 (0.22-0.30)	560.79	60	0.11 (0.08-1.38)
30-34	139.05	78	0.56 (0.44-0.70)	126.89	52	0.41 (0.31-0.54)
35-39	114.82	114	0.99 (0.82-1.19)	108.21	122	1.12 (0.94-1.35)
40-44	130.28	191	1.47 (1.27-1.69)	124.54	180	1.44 (1.24-1.67)
45-49	142.04	349	2.46 (2.21-2.73)	134.63	189	1.41 (1.21-1.62)
50-54	130.31	330	2.53 (2.27-2.82)	118.59	239	2.01 (1.77-2.29)
55-59	75.46	402	5.33 (4.82-5.87)	68.12	376	5.53 (4.98-6.11)
60-64	84.09	464	5.52 (5.03-6.04)	80.14	281	3.50 (3.11-3.94)
65-69	60.7	298	4.91 (4.37-5.50)	58.91	183	3.11 (2.67-3.59)
70-74	40.6	220	5.43 (4.73-6.18)	40.22	115	2.86 (2.36-3.43)

75-79	29.84	95	3.20 (2.58-3.89)	31.05	47	1.52 (1.11-2.01)
80-84	19.5	26	1.34 (0.87-1.95)	22.95	14	0.61 (0.33-1.02)
>=85	13.29	7	0.50 (0.21-1.09)	19.35	1	0.05 (0.01-0.29)
Area[a]						
East	748.36	1524	2.04 (1.94-2.14)	701.69	1049	1.49 (1.41-1.59)
North	82.91	95	1.15 (0.93-1.40)	79.65	56	0.70 (0.53-0.91)
North-East	72.84	144	1.98 (1.67-2.33)	76.27	119	1.56 (1.29-1.87)
North-West	74.84	117	1.56 (1.29-1.87)	70.22	54	0.77 (0.58-1.00)
South-Central	418.71	675	1.61 (1.49-1.74)	360.47	471	1.31 (1.19-1.43)
South-West	219.29	186	0.85 (0.73-0.98)	206.07	111	0.54 (0.44-0.65)

[a] Area was defined as in Table 1.

eTable 5 Sensitivity analysis results for prevalence and incidence (Units: /100 000 population for prevalence; /100 000 person-years for incidence)

	Including all suspected MM cases	Using only observed cases[a]	Using the observed number of MM cases in each province as weights	Excluding the top 10% of the provinces along the absence of the diagnosis[b]
Prevalence				
Overall	6.95 (5.81-8.08)	3.47 (2.73-4.20)	8.29 (8.22-8.36)	7.00 (5.71-8.29)
Male	7.97 (6.58-9.35)	3.96 (3.16-4.76)	9.25 (9.15-9.34)	8.05 (6.52-9.58)
Female	5.85 (4.90-6.79)	2.94 (2.25-3.63)	7.33 (7.24-7.43)	5.89 (4.79-6.98)
Incidence				
Overall	1.62 (1.29-1.94)	0.85 (0.70-0.99)	1.68 (1.62-1.73)	1.57 (1.25-1.90)
Male	1.86 (1.49-2.22)	0.95 (0.79-1.11)	1.86 (1.78-1.94)	1.78 (1.42-2.14)
Female	1.31 (1.02-1.61)	0.69 (0.55-0.83)	1.52 (1.44-1.60)	1.30 (1.00-1.60)

[a] Known to be an underestimation of rates.

[b] Shandong and Xinjiang were excluded.
